# A Modified Dynamic Evolving Neural-Fuzzy Approach to Modeling Customer Satisfaction for Affective Design

**DOI:** 10.1155/2013/636948

**Published:** 2013-12-09

**Authors:** C. K. Kwong, K. Y. Fung, Huimin Jiang, K. Y. Chan, Kin Wai Michael Siu

**Affiliations:** ^1^Department of Industrial and Systems Engineering, The Hong Kong Polytechnic University, Kowloon, Hong Kong; ^2^Department of Electrical and Computer Engineering, Curtin University of Technology, Perth, WA 6845, Australia; ^3^School of Design, The Hong Kong Polytechnic University, Kowloon, Hong Kong

## Abstract

Affective design is an important aspect of product development to achieve a competitive edge in the marketplace. A neural-fuzzy network approach has been attempted recently to model customer satisfaction for affective design and it has been proved to be an effective one to deal with the fuzziness and non-linearity of the modeling as well as generate explicit customer satisfaction models. However, such an approach to modeling customer satisfaction has two limitations. First, it is not suitable for the modeling problems which involve a large number of inputs. Second, it cannot adapt to new data sets, given that its structure is fixed once it has been developed. In this paper, a modified dynamic evolving neural-fuzzy approach is proposed to address the above mentioned limitations. A case study on the affective design of mobile phones was conducted to illustrate the effectiveness of the proposed methodology. Validation tests were conducted and the test results indicated that: (1) the conventional Adaptive Neuro-Fuzzy Inference System (ANFIS) failed to run due to a large number of inputs; (2) the proposed dynamic neural-fuzzy model outperforms the subtractive clustering-based ANFIS model and fuzzy *c*-means clustering-based ANFIS model in terms of their modeling accuracy and computational effort.

## 1. Introduction

Today, manufacturers face a highly competitive environment, as marketing becomes global and large amount of choices are available in the market. Customers consider functionality, ease-of-use, and reliability as product requirements, they but equally consider intangible and emotional aspects such as the novelty, personality, aesthetics, and style of products [1, 2]. Therefore, design for performance and design for usability can no longer guarantee a competitive advantage [3]. Products with highly affective designs can attract customers, influence their choices and preferences, and gain their loyalty while giving them the joy of use [4–6]. Thus, affective design is essential to identify, measure, analyze, and understand the relationships between the affective values of the customer domain and the perceptual design parameters in the design domain [7, 8]. Furthermore, affective design provides decision support for the design optimization process, such that appealing products can be successfully developed by satisfying the emotional needs of the target customers [9, 10].

Affective design in industries still heavily relies on the experience and intuition of designers, who may not fully recognize or perceive the affective values of their customers. Thus, cognitive gaps in design appreciation always exist between designers and customers [1, 11]. Although the customers' emotional and implicit needs can be obtained and collected from focus groups and in-depth interviews, the collected information is subjective and qualitative because the sampling size is limited. Given the importance of affective design, previous studies attempted to adopt quality function deployment and robust design approaches to affective design [12, 13]. However, these approaches are incapable of dealing with the subjective aspects of affective design [14].

Affective relationships are mostly nonlinear because of the inconsistencies in the customer survey data and their nonlinear relationships with design attributes [13]. Given the fuzzy and non-linear behavior of affective modeling, statistical methods based on the assumptions of simple linear relationships and normal distributions may not be capable of modeling affective values [15]. Studies on affective design have attempted to apply computational intelligence techniques, specifically artificial neural networks, to deal with the ambiguity of affective data and the nonlinearity of affective modeling [16]. Although these approaches are capable of modeling the non-linear relationships between parameters, they generate models that are implicit, that is, black-box models. Thus, the analysis of the behavior of these relationships remains difficult.

A hybrid approach, named the neural fuzzy (NF) model, has been used to model the relationships between design attributes and affective responses of customers [17–19]. The NF model combines the capability of fuzzy logic to generate the linguistic representation of knowledge and the adaptive learning capability of artificial neural networks for the automatic generation and optimization of a fuzzy inference system. The model is formed by complex networks that consist of significant numbers of hidden nodes, linkage weights, and fuzzy membership functions, among others. Based on these complex networks, the model effectively captures the highly non-linear relationships between the design attributes of a new product and its satisfaction of affective dimensions. Furthermore, the networks of the NF models overcome the limitations of artificial neural networks by obtaining explicit information that is useful for affective design.

A large number of design attributes may need to be studied in an affective design. Furthermore, the initialization of customer satisfaction models is based on a limited amount of customer surveys, which are collected by a focus group. When new survey data is collected, the customer satisfaction model will need to be updated with respect to the data from the new survey. The network structures of NF models tend to be too complex and too cumbersome. Thus, the effectiveness of these networks is questionable for modeling customer affections that have multiple dimensions and require updates. In this paper, a modified dynamic evolving neural-fuzzy inference systems (DENFIS) approach is proposed to overcome the limitations of the existing NF approaches with respect to modeling affective relationships with high dimensions and updating models with different batches of survey data. The effectiveness of the proposed approach is demonstrated using a case study of affective design for mobile phones.

## 2. Dynamic Evolving Neurofuzzy Inference Systems (DENFIS)

Adaptive neural fuzzy inference systems (ANFIS) have been used [20] to model customer satisfaction, where the inputs and the outputs of the NF models represent the design attributes and the satisfaction values of the new product, respectively. However, the fixed structure generated by ANFIS hinders its adaption to new data sets. In addition, ANFIS cannot be used for high dimensional problems [21], because the complexity of the dynamic NF models generated by ANFIS typically grows exponentially with the number of input attributes. The number of rule nodes generated is equal to ∏_*j*=1_^*J*^ *N*_MF,*j*_, where *N*_MF,*j*_ is the number of membership functions (MF) for the *j*th design attribute and *J* is the number of design attributes of the customer affection model. For instance, the structure of the NF model contains 6,561 (= 3^8^) rule nodes if there are eight inputs, and each input contains three MFs. Excessive inputs and MFs could cause computational difficulties because of the insufficient machine memory [22]. Therefore, ANFIS is unsuitable for generating customer affection models with high dimensions of inputs. The DENFIS approach is considered in this research to develop dynamic NF models for modeling customer satisfaction for affective design.

### 2.1. Conventional DENFIS

The conventional DENFIS can be used to generate a streamlined model in NF form for affective design, which cannot be achieved by the modified DENFIS [23, 24], in which a reasonable number of fuzzy rule-based models are generated. Conventional DENFIS is a fast, one-pass, online incremental clustering algorithm for generating NF models for affective design; this system generates input spaces using the evolving clustering method [23, 25]. The survey data on affective values are partitioned by the system into clusters, which are specified by their centers and radii. Thus, the number of created clusters is self-determined based on a threshold value, *D*_thr_, which controls the maximum distance between a data point of a cluster and the cluster center and acts as a constraint for updating the radii of the clusters.

In the clustering process, the evolving clustering method starts by initializing the first cluster *C*_1_ with the first data survey data regarding affective values (*Z*_1_); that is, *k* = 1, where *k* is the number of clusters created by the evolving clustering method. When new data on affective values (*Z*_*n*_) is presented, its distance to the cluster centre (*c*_*k*_), of each existing cluster (*C*_*k*_) is computed, where *k* = 1,2,…, *K*, and *n* > 1. Therefore, the distance *D*′ between the *n*th survey data (*Z*_*n*_) and its closest cluster (*C*′) can similarly be found. The new point belongs to the cluster *C*′, and an update does not occur when *D*′ < *r*′, where *r*′ denotes the current radius of the cluster *C*′. If *D*′ < *D*_thr_, the centre *c*′ and radius *r*′ of the closest cluster *C*′ are updated [23]. Otherwise, a new cluster *C*_*K*+1_ is created at the *n*th survey data *Z*_*n*_, such that *K* = *K* + 1. Consequently, the newly collected survey data can be dynamically grouped by applying the evolving clustering method. This step facilitates the formulation of the optimal number of local models for effective modeling substantial data.

After determining the partitions of the input space using the clustering process, a set of fuzzy rules are then created to represent the cluster. In the rule antecedents, fuzzy numbers are used to represent the design attributes of the new product. In the rule consequents, first-order linear models are used to represent the affective values of the new product. The fuzzy rules are expressed as follows:

(1) if  x1  is  MF11  and  x2  is  MF21  and…and  xJ  is  MFJ1, then  y  is  f1(x1,x2,…,xJ);if  x1  is  MF12  and  x2  is  MF22  and…and  xJ  is  MFJ2, then  y  is  f2(x1,x2,…,xJ)⋮if  x1  is  MF1K  and  x2  is  MF2K  and…and  xJ  is  MFJK, then  y  is  fK(x1,x2,…,xJ),

where *x*_*j*_ is MF_*jk*_, *j* = 1,2,…, *J*, and *k* = 1,2,…, *K*, MF_*jk*_ denotes a Gaussian MF used for the fuzzification of the design attributes of new products, *J* is the number of design attributes of the new products, *K* is the number of clusters generated by the evolving clustering method, and *f*_*k*_(*x*_1_, *x*_2_, …, *x*_*J*_) denotes the function of a first-order linear model for affective design.

The input space of each fuzzy rule is fuzzified based on the clustering results of the evolving clustering method. For each cluster, the cluster centre *c*_*k*_ and radius *r*_*k*_ are assigned to the Gaussian MF, which is defined as

(2) $$\begin{array}{c} \text{Gaussian}\;\;\text{M}{\text{F}}_{jk}=\alpha \cdot \exp\left[-\frac{\left(x_{j}-c_{jk}\right)}{2r_{jk}}\right]. \end{array}$$

For the rule consequent of each fuzzy rule, a first-order linear model is developed based on a weighted recursive least square, which uses a forgetting factor in the conventional DENFIS [23, 25]. To represent the satisfaction values of affective dimensions of the new product, the first-order linear model is represented by the following linear polynomial:

(3) $$\begin{array}{c} y=\;\beta_{0}+\beta_{1}x_{1}+\beta_{1}x_{2}+\cdots +\beta_{J}x_{J}. \end{array}$$

For each rule, the *u* data instances {[*x*_1_^*i*^, *x*_2_^*i*^,…, *x*_*j*_^*i*^,…, *x*_*J*_^*i*^], *y*_*i*_} with *i* = 1,2,…, *u*, belong to the same cluster. These instances are intended to be the initial training data for the recursive least squares. For the initial linear model, the regression coefficients of **β** = [*β*_0_,  *β*_1_,  *β*_2_,  *β*_*j*_,  *β*_*J*_]^*T*^ are calculated by applying the weighted least squares estimator using the following formulas:

(4) $$\begin{array}{c} P={\left(A^{T}WA\right)}^{-1},\\ \beta =PA^{T}Wy,\; \end{array}$$

where

(5) $$\begin{array}{c} A=\left(\begin{array}{c} \begin{array}{ccccccc} 1 & x_{1}^{1} & x_{2}^{1} & \cdots & x_{j}^{1} & \cdots & x_{J}^{1}\\ 1 & x_{1}^{2} & x_{2}^{2} & \cdots & x_{j}^{2} & \cdots & x_{J}^{2}\\ \vdots & \vdots & \vdots & \ddots & \vdots & \ddots & \vdots \\ 1 & x_{1}^{i} & x_{2}^{i} & \cdots & x_{j}^{i} & \cdots & x_{J}^{i}\\ \vdots & \vdots & \vdots & \ddots & \vdots & \ddots & \vdots \\ 1 & x_{1}^{u} & x_{2}^{u} & \cdots & x_{j}^{u} & \cdots & x_{J}^{u} \end{array} \end{array}\right),\\ y={\left[\begin{array}{cccccc} y_{1} & y_{2} & \cdots & y_{i} & \cdots & y_{u} \end{array}\right]}^{T},\\ W=\left(\begin{array}{c} \begin{array}{cccccc} \omega_{1} & 0 & \cdots & 0 & \cdots & 0\\ 0 & \omega_{2} & \cdots & 0 & \cdots & 0\\ \vdots & \vdots & \ddots & \vdots & \ddots & \vdots \\ 0 & 0 & \cdots & \omega_{i} & \cdots & 0\\ \vdots & \vdots & \ddots & \vdots & \ddots & \vdots \\ 0 & 0 & \cdots & 0 & \cdots & \omega_{u} \end{array} \end{array}\right) \end{array}$$

of which *ω*_*i*_ is (1 − *D*_*i*_) with *i* = 1, 2,…, *u*, and *D*_*i*_ is the distance between the *i*th survey data regarding affective values and the corresponding cluster centre.

The regression coefficient **β** and inverse matrix **P** are used as the initial values for the future recursive calls, where **β**_*u*_ and **P**_*u*_ represent the regression coefficient and the inverse matrix, respectively, at the *u*th iteration using the least squares. When new survey data regarding affective values is fed, **β**_*u*+1_ is updated based on the following equations:

(6) $$\begin{array}{c} \beta_{u+1}=\beta_{u}+\omega_{u+1}P_{u+1}a_{u+1}\left(y_{u+1}-a_{u+1}^{T}\beta_{u}\right),\\ P_{u+1}=\frac{1}{\lambda }\left(P_{u}-\frac{w_{u+1}P_{u}a_{u+1}a_{u+1}^{T}P_{u}}{\lambda +a_{u+1}^{T}P_{u}a_{u+1}}\right), \end{array}$$

where *λ* is a forgetting factor such that 0 < *λ* ≤ 1, and

(7) $$\begin{array}{c} a_{u+1}^{T}=\left[\begin{array}{ccccc} 1 & x_{1}^{u+1} & x_{2}^{u+1} & \begin{array}{ccc} \cdots & x_{j}^{u+1} & \cdots \end{array} & x_{J}^{u+1} \end{array}\right]. \end{array}$$

**Figure 1:**
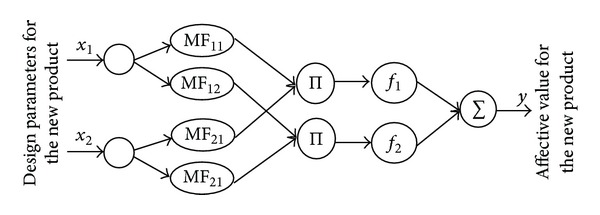
A five-layer structure of the dynamic NF model (*J* = 2, *K* = 2) for affective design.

Finally, a dynamic NF model can be created based on the set of fuzzy rule-based models that are generated using the evolving clustering method and the recursive least squares. As an illustration, Figure 1 shows a typical structure of a five-layer NF model with two design attributes for a new product, *x*_1_ and *x*_2_, as well as one affective dimension for the new product, *y*. Subsequently, the back propagation (BP) algorithm can be used to further optimize the fuzzy MF of the dynamic NF model for affective design.

### 2.2. Modified DENFIS for Affective Design

**Figure 2:**
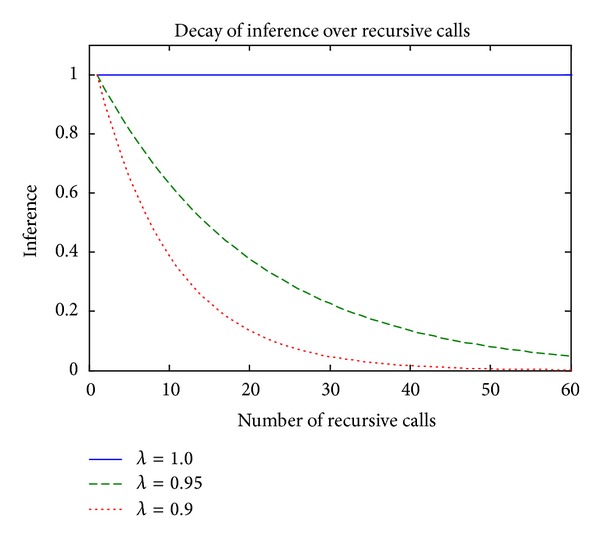
Decay of inference with a constant forgetting factor.

It can be noted that the conventional DENFIS can only generate a dynamic NF model in the single-feed form, where only a single instance of survey data can be used to update the dynamic NF model. The influence of each data instance is typically decayed by applying the recursive least squares with a constant forgetting factor *λ*, as shown in Figure 2.

For affective design, some companies may conduct surveys several times over a period of time to obtain more accurate data on customer affection towards the designs of products. The respondent evaluates the design profiles of *N* product samples. The design profile of the *n*th product sample can be represented as *X*(*n*) = [*x*_1_^*n*^, *x*_2_^*n*^,…, *x*_*j*_^*n*^,…, *x*_*J*_^*n*^], where *n* = 1,2,…, *N*. Subsequently, *N* customer affections are obtained from the survey which is participated in at time *t*. The affective data set collected at time *t* is given by

(8) $$\begin{array}{c} Y\left(t\right)=\left[y_{1}\left(t\right),y_{2}\left(t\right),\ldots ,y_{N}\left(t\right)\right], \end{array}$$

where *y*_*n*_(*t*) denotes the affective rating acquired from the respondent towards the *N* product samples after conducting the survey at the *t*th period. Thus, the *N* survey data sets are available for updating the NF model for affective design.

A modified DENFIS is proposed to process a batch of *N* survey data sets for each update process, where the forgetting factor *λ* is a variable instead of a constant, such that the decay effect can be controlled by varying the value of *λ*. Typically, *λ* < 1 is used for the recursive least squares so the fresh data set can exert more influence than the previous data set during the recursive calls. By contrast, previous and current data sets are treated equally by the recursive least squares if *λ* = 1. The forgetting factor value is switched to update data sets from different periods of time when the modified DENFIS is performing the incremental learning process.

**Figure 3:**
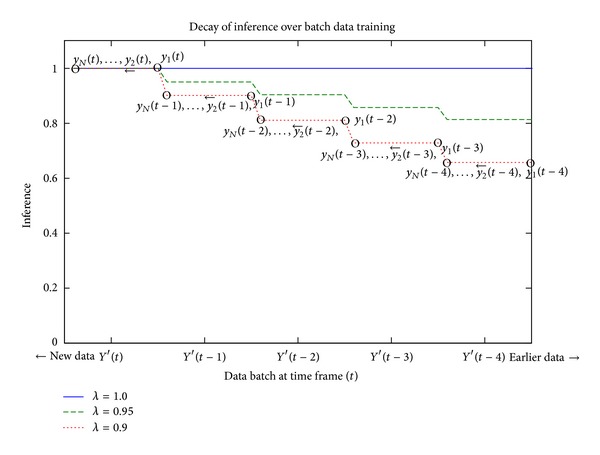
Decay of inference by the modified DENFIS.

The modified DENFIS was developed to decrease the influence of survey data sets by batch over time. When the survey data set *Y*(*t*) is available to the model, its influence is greater than that of the previous data set *Y*(*t* − 1). During the incremental learning process, *y*_1_(*t*), *y*_2_(*t*),…, *y*_*N*_(*t*) are partitioned into clusters based on the evolving clustering method. For each cluster, the first-order model is updated by the data subset using the recursive least squares. When the first data instance of the subset, such as *y*_1_(*t*), is proceeded by new survey data, *λ* < 1 is set to exert the decay effect on previous data batches, which are compiled as the matrices *β*_*t*−1_ and *P*_*t*−1_. *β*_*t*−1_ and *P*_*t*−1_ have been obtained from the previous recursive least squares in the previous data batch *Y*(*t* − 1). For the remaining data in the data subset, *λ* = 1 is set to suspend the decay during the training sequence from *y*_1_(*t*) to *y*_*N*_(*t*). Thus, the previous data batch fades out when the current data batch is processed with the same influence. This staircase decay is shown in Figure 3.

**Figure 4:**
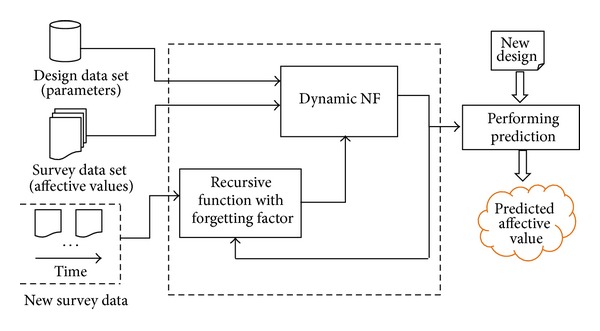
A dynamic NF model for affective design.

Figure 4 summarizes the architecture of the modified DENFIS for affective design, where the dynamic NF model enables faster incremental learning by applying the evolving clustering method and the recursive least squares. The modified DENFIS uses the recursive least squares with the variable forgetting factor to update the dynamic NF model by adapting the new data sets.

## 3. Case Study of Affective Design for Mobile Phones

A case study of the affective design for mobile phones is used to investigate the effectiveness of the proposed approach to modeling affective relationships. The case study mainly involves a survey and the implementation of the proposed approach for the affective design of mobile phones. The modified DENFIS was implemented using the MATLAB programming language.

**Figure 5:**
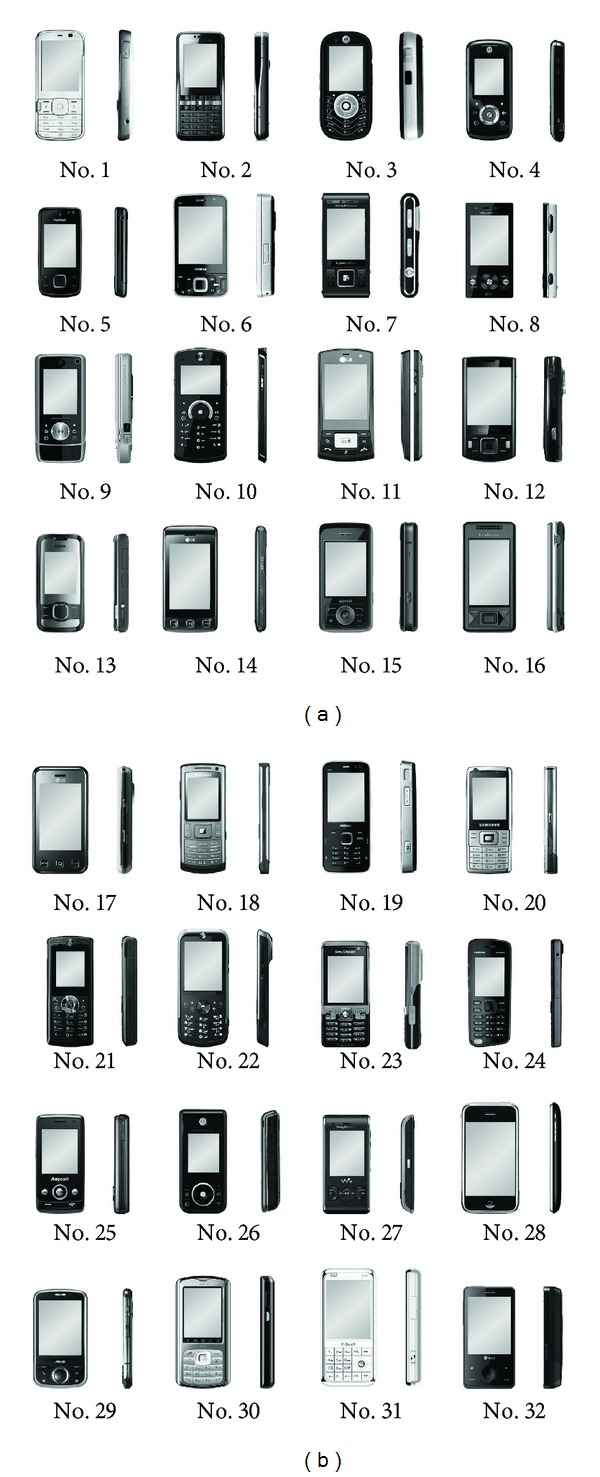
The 32 mobile phone image samples used in the case study.

**Figure 6:**
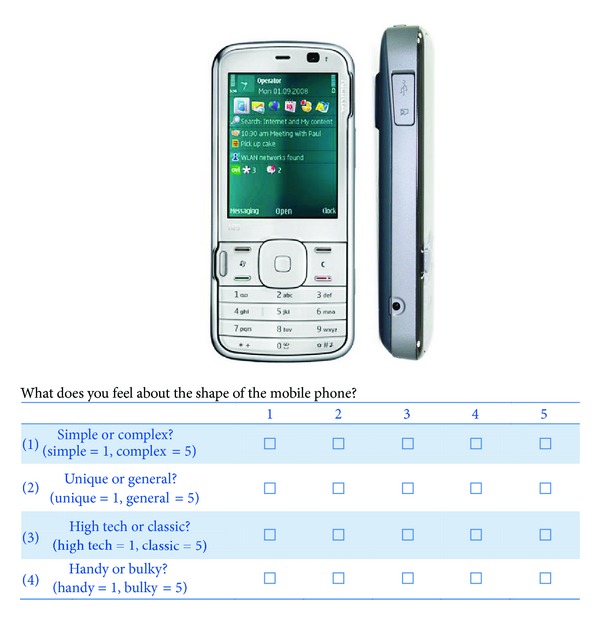
The questionnaire format for each mobile phone.

The survey was conducted using questionnaires. The affective assessments of customers on 32 image samples of mobile phones were determined using four affective dimensions: “simplicity,” “uniqueness,” “high tech,” and “handiness.” The front and side views of the 32 mobile phone samples are presented in Figure 5. A total of 34 respondents filled out the questionnaires and indicated their feelings towards the product images of each sample using a five-point Likert scale, which is illustrated in Figure 6.

The morphological approach was adopted to define the design space of the product form for the mobile phones. The design composition and the possible design solutions with simple and graphical notations were feasibly depicted. Eight design attributes (from *A*_1_ to *A*_8_) were defined to describe the product forms of mobile phones, which included the top shape, bottom shape, function button shape, layout, length, width ratio, thickness, and border width. The first four design attributes are categorical, and the remaining four attributes are quantitative. The categorical attributes contain three to five options. The attributes and their options are listed in a design table (Table 1) for the product form of the mobile phones. Based on the design table, the design profile for each sample was identified, and the values of design attributes were measured.

### 3.1. Implementation of the Dynamic NF Model

An initial dynamic NF model for modeling affective relationships was developed using the proposed DENFIS when the initial survey data sets were collected from the first seventeen respondents. The dynamic NF model was then sequentially updated using the survey data sets collected from the subsequent respondents. The forgetting factor *λ* = 0.95 was set to produce a slight decay of inference. Two dynamic NF models were developed; one involved BP training, whereas the other did not.

**Figure 7:**
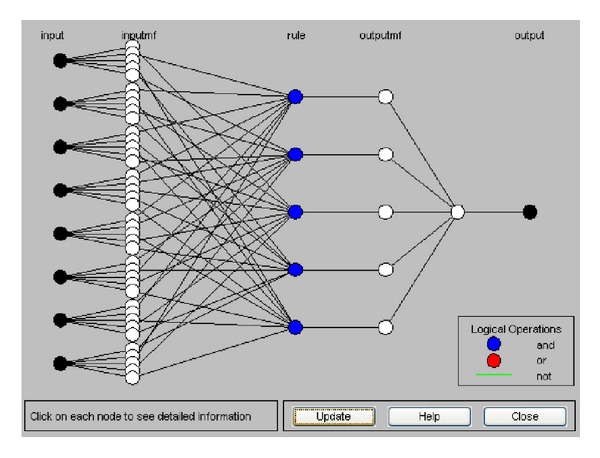
Structure of the developed dynamic NF model for the affective design of mobile phones.

**Figure 8:**
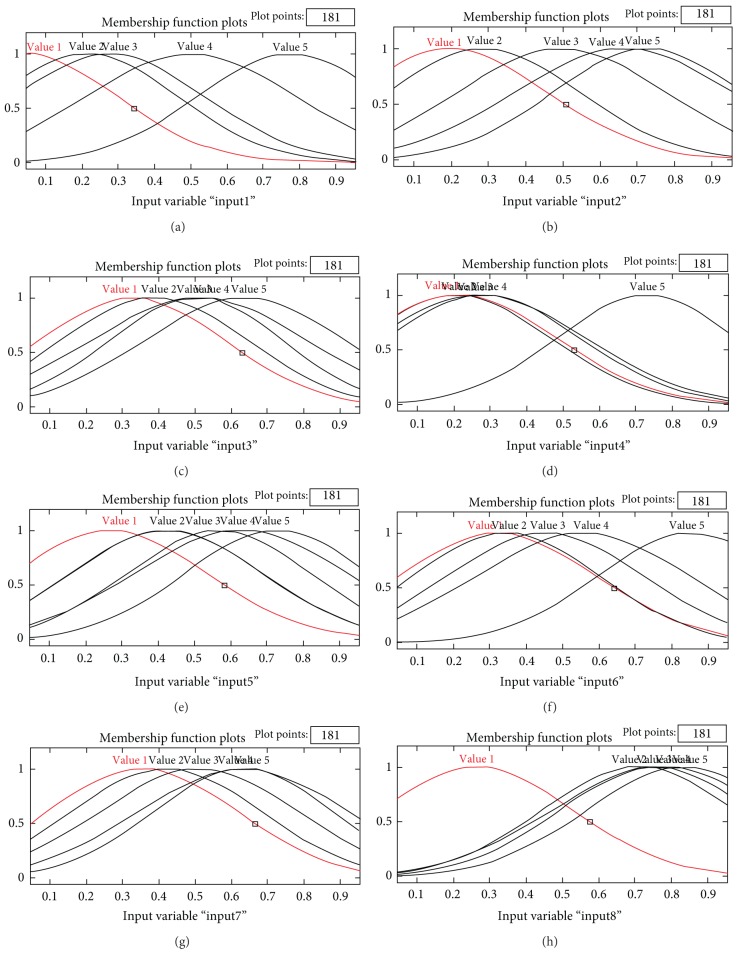
Fuzzy membership functions of the affections of mobile phone: (a) top shape, (b) bottom shape, (c) function button shape, (d) layout, (e) body length, (f) body thickness, and (g) border width.

The structure of the dynamic NF model that was generated using the DENFIS is shown in Figure 7, where eight nodes in the input layer represent the eight design attributes of mobile phone design. Each node in the input layer links with five MFs in the input layer, and the MFs are engaged to the five nodes in the rule layer. All the nodes in the three layers can be represented by a set of fuzzy rules that are formulated from the generated clusters of the evolving clustering method.  Algorithm 1 presents these fuzzy rules, where each fuzzy rule can be considered as a local fuzzy model. Each local fuzzy model consists of fuzzy domains governed by fuzzy MFs for the design attributes, as shown in Figure 8. After inputting design attribute settings of mobile phones, the resulting local models were aggregated to determine satisfaction values of the affective dimensions for mobile phones.

### 3.2. Prediction Performance of the Dynamic NF Models

The leave-one-out cross-validation is performed to evaluate the prediction performance of the proposed dynamic NF model for the affective design of mobile phones. Data on the 32 mobile phone samples are randomly partitioned into training and test sets. The training set consists of 31 samples, while the test set contains only 1 sample. The cross-validation test involves all 32 folds, where each fold underwent the replacement of the training and test sets during the training process. Hence, each sample can be used as a test set only once.

The prediction performance of the two dynamic NF models that were generated by DENFIS (either with or without BP training) are compared with those generated by the common ANFIS approaches, namely, the subtractive clustering- (SC-) based ANFIS approach and the fuzzy *c*-means clustering- (FCM-) based ANFIS approach [22]. In this case study, a fully structured ANFIS was developed for the affective design. However, its training process could not be completely done because its structure generated by ANFIS approach contained more than 10935 hidden nodes, which was too complex and also required a large amount of computational memory for execution. When using the SC-based ANFIS approach and the FCM-based ANFIS approach, the hidden nodes are selectively built into the NF models based on the partitioning results of the SC and FCM clustering approaches, respectively. Therefore, a much less computational memory is required as compared with the fully structured ANFIS approach. Using the same training data sets, four NF models are generated based on the four approaches, namely, DENFIS with BP training, DENFIS without BP training, SC-based ANFIS, and FCM-based ANFIS. The generated NF models are then applied to estimate satisfaction values of the affective dimensions using the test data sets. Subsequently, the performances of the four NF models are compared based on the average root mean square error (RMSE) and the computational time, where the average RMSE is defined as follows:

(9) Average  RMSE=∑RMSE of test samplesTotal number of test samples in the cross-validation test.

**Figure 9:**
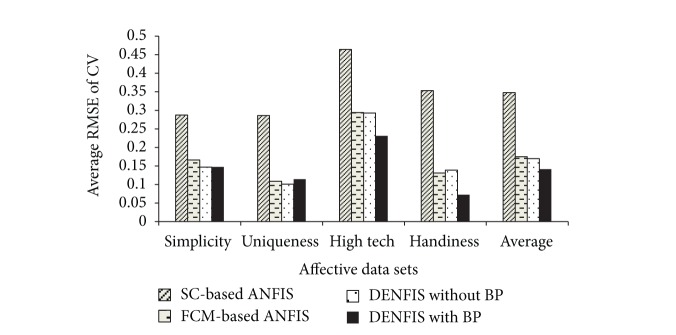
Histogram of the average RMSE of the NF models.

The results of the cross-validation tests for the four affective dimensions: “simplicity,” “uniqueness,” “high tech,” and “handiness,” are shown in Tables 2, 3, 4, and 5, respectively, whereas Figure 9 summarizes the results obtained by the four approaches. The test results for “simplicity,” “uniqueness,” and “high tech” reveal that the test errors when using the DENFIS without BP training are smaller than those obtained when using the FCM-based models. Moreover, the DENFIS with BP training outperforms the FCM-based ANFIS models in terms of its prediction performance. DENFIS with BP training significantly reduces the errors of the dynamic NF model for “high tech” and “handiness,” because BP training fine-tunes membership functions and improves the prediction accuracy of the dynamic NF models. The results shown in Tables 2 and 3 reveal that the errors of the NF models generated by DENFIS with BP training are slightly larger than those without BP training because overfitting may occur when BP training is used. However, the NF models that were generated by DENFIS with BP training obtain the best overall prediction performance as compared with the other three approaches.

Apart from the prediction performance, the test results show that the two DENFIS approaches are more computationally efficient than the other two ANFIS approaches. The computational time of the incremental training for the two DENFIS approaches is approximately 0.018 s, which is about 18% to 29% less than that of the two ANFIS approaches.

## 4. Conclusion

This paper proposes a dynamic evolving NF approach to modeling the relationships between design attributes and affective satisfaction which is based on collected customer survey data. The proposed approach is able to address the high nonlinearity and fuzziness of the modeling of the affective relationship, which cannot be addressed effectively by the commonly used statistical methods. The proposed model likewise addresses the two important aspects that cannot be handled by the existing NF models: (i) the existing NF models cannot be used to model the affective relationships which involve a large number of inputs; and (ii) the existing NF models cannot adapt to customers' affective preferences effectively that are obtained from newly collected survey data. The effectiveness of the proposed approach to modeling customer satisfaction for affective design is demonstrated using a case study of the affective design of the mobile phones. Results of the validation tests show that the dynamic NF model displays better prediction performance and shorter processing time as compared with the existing NF models including the SC-based and FCM-based ANFIS models. The performance of the dynamic NF models with BP training is the best among the NF models in the cross-validation tests. In addition, the dynamic NF models reduce the computational time by 18% to 29% as compared with the other two models. The fast incremental training process of the dynamic NF model enables long-term updates and maintenance of these models. Future work could study the generated models based on the proposed approach to determine the optimal setting of the design attributes for affective design.

## Figures and Tables

**Algorithm 1:**
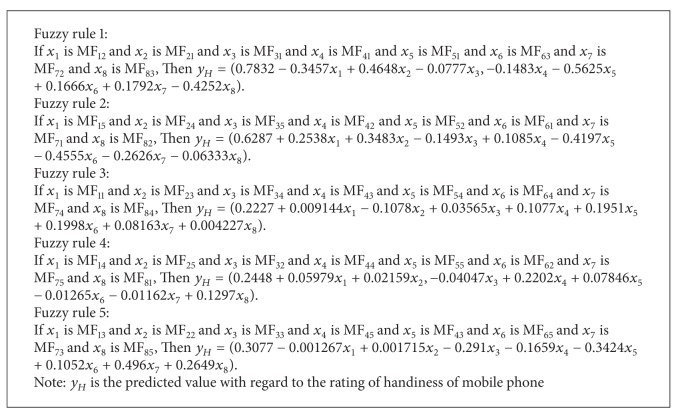
A set of fuzzy rules engaged with the dynamic NF model for the affective design of mobile phones.

**Table 1:** Design table for the product form of the mobile phones.

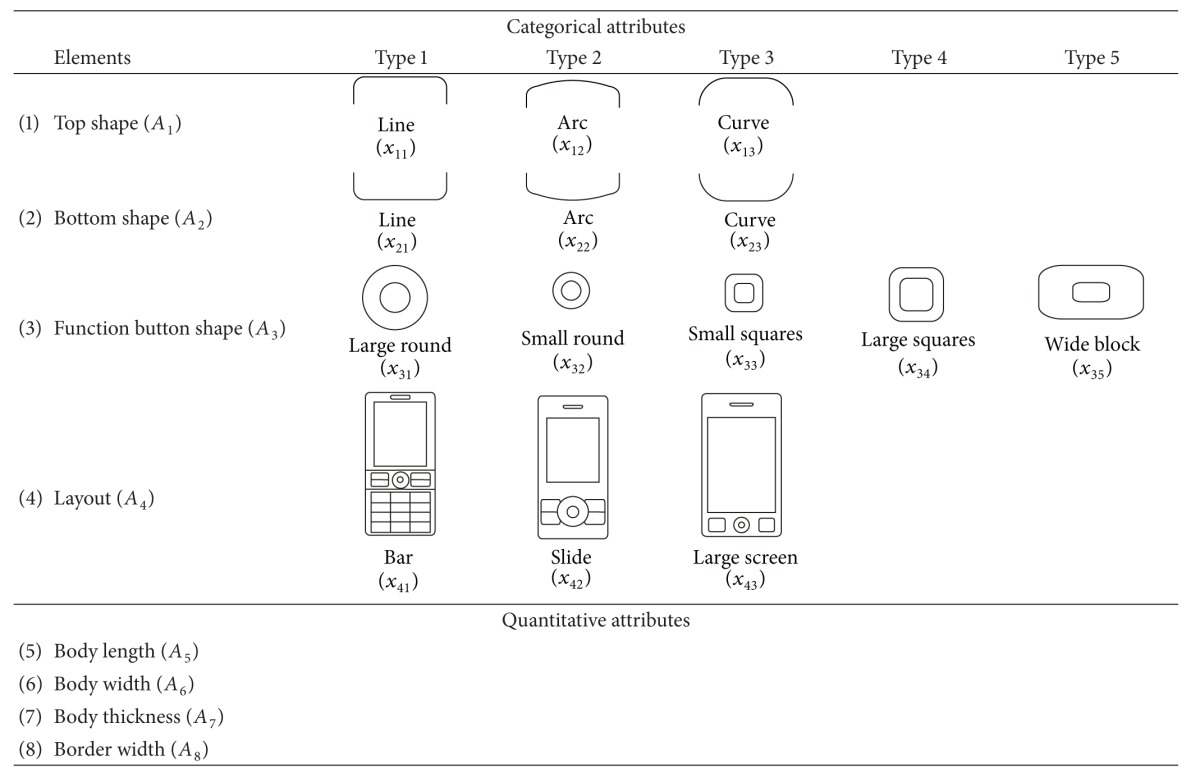

**Table 2:** Test performance of the NF models (simplicity).

Models	Average RMSE	Average computational time*/s
SC-based ANFIS	0.2874	0.6921
FCM-based ANFIS	0.166	0.6157
DENFIS without BP	0.147	0.4991
DENFIS with BP	0.1474	

*Average computational time per update of dynamic NF models is 0.01848 s.

**Table 3:** Test performance of the NF models (uniqueness).

Models	Average RMSE	Average computational time*/s
SC-based ANFIS	0.2859	0.692
FCM-based ANFIS	0.1086	0.606
DENFIS without BP	0.101	0.4876
DENFIS with BP	0.1143	

*Average computational time per update of dynamic NF models is 0.01806 s.

**Table 4:** Test performance of the NF models (high tech).

Models	Average RMSE	Average computational time*/s
SC-based ANFIS	0.464	0.6048
FCM-based ANFIS	0.2941	0.6887
DENFIS without BP	0.2927	0.493
DENFIS with BP	0.2312	

*Average computational time per update of dynamic NF models is 0.01826 s.

**Table 5:** Test performance of the NF models (handiness).

Models	Average RMSE	Average computational time*/s
SC-based ANFIS	0.3533	0.6859
FCM-based ANFIS	0.1312	0.6277
DENFIS without BP	0.1387	0.5047
DENFIS with BP	0.07244	

*Average computational time per update of dynamic NF models is 0.01869 s.
